# Risk factors associated with anaemia among pregnant women in the Adaklu District, Ghana

**DOI:** 10.3389/fgwh.2023.1140867

**Published:** 2024-02-15

**Authors:** Eric Tettegah, Thomas Hormenu, Nancy Innocentia Ebu-Enyan

**Affiliations:** ^1^ Department of Health, Physical Education and Recreation, Faculty of Science and Technology Education, University of Cape Coast, Cape Coast, Ghana; ^2^ Department of Adult Health, School of Nursing and Midwifery, University of Cape Coast, Cape Coast, Ghana

**Keywords:** anaemia, pregnant women, factors, barriers, Ghana

## Abstract

**Background:**

Anaemia during pregnancy is a major public health concern in both advanced and less-developed countries including Ghana. The prevalence of anaemia in Ghana has a serious repercussion on the country's social and economic development. This prevalence has been linked to various factors, including educational level and occupational status.

**Methods:**

A prospective study was conducted to investigate the factors influencing anaemia among 150 pregnant women, aged 15–49 years, who attended antenatal clinics in the Adaklu District of the Volta region of Ghana. Haemoglobin levels were assessed in the first, second, and third trimesters, and a questionnaire was also used to collect data on demographic information and barriers to maintaining haemoglobin levels. The data were analysed using frequencies, percentages, and binary logistic regression.

**Results:**

The prevalence of pregnancy anaemia in the district was 78.5%. The study found that 92% and 8% of pregnant women had excellent and good knowledge on anaemia in pregnancy, respectively. The study also identified several barriers to maintaining an appropriate haemoglobin level during pregnancy, such as long distances to healthcare facilities, non-intake of antimalarial drugs, and lack of nutritious meals. Finally, the study found that low education level, number of pregnancies, and number of children a woman had were significant determinants of anaemia during pregnancy in the district.

**Conclusion:**

The findings of the study suggest that targeted interventions are needed to reduce the burden of anaemia during pregnancy in the district. These interventions should address the social and environmental determinants of anaemia during pregnancy, such as improving access to healthcare facilities and promoting healthy eating habits. In addition, interventions that address social determinants of health, such as education and occupation, may be effective in reducing the burden of anaemia during pregnancy in the district.

## Introduction

Anaemia during pregnancy is a significant issue that affects both developed and developing countries and has significant social and economic implications for the affected nations (1). Anaemia is defined as a decrease in the concentration of haemoglobin below a specific threshold, which depends on various factors such as age, gender, physiological status, smoking habits, and altitude of the population being evaluated. According to the World Health Organization (WHO), anaemia during pregnancy is defined as a haemoglobin concentration less than 11 g/dl at sea level (2). Kefiyalew (3) also share the view that, during pregnancy, a haemoglobin level below 11 g/dl of human blood is referred to as anaemia. The prevalence of anaemia among pregnant women varies because of the differences in socio-economic status, lifestyles, and health seeking behaviours across diverse cultures during pregnancy (4). Globally, anaemia adversely affects billions of people. About 32.4 million (38%) of the pregnant women had anaemia, with a higher prevalence of 49% in South East Asia (5).

Anaemia affects almost a billion women in their fertility age around the world (6). In 2011, anaemia affects 38% of pregnant women aged 15–49 years and 29% of non-pregnant women globally. However, a higher prevalence was reported in South Asia, Central Asia, and West Africa. Many countries and international bodies have implemented measures that are geared towards preventing and controlling the devastating impacts of maternal anaemia. The periodic data collection and identification of the cause of maternal anaemia will help assess the outcome of the interventions being implemented and the strategies being used by the government to control and prevent maternal anaemia (7). The WHO estimates that 52% of pregnant women in developing countries are anaemic, as against to 23% in the developed world (8). Research conducted in Africa indicated increasing cases of maternal anaemia ranging from 41% to 83% recorded in different geographical settings (9). The Family Health Division (FHD) of the Ghana Health Service Annual Report (10) revealed that the Volta Region had the highest prevalence (50%) of anaemia among pregnant women in their fertility age (15–49). The high rate of maternal anaemia in sub-Saharan Africa (SSA) was a result of most women commencing pregnancy with insufficient iron level and vitamins in the body. Again, the high prevalence of maternal anaemia can be associated with a lack of family planning, poor dietary intake, blood loss during menstruation, and persistent infections.

Socio-economic factors, negative lifestyles, and different cultures have been explicitly implicated as the determinants of high cases of maternal anaemia in SSA (11). In Ghana, research indicates that about 50% of all anaemia cases are as a result of iron deficiency (12). On the contrary, governmental policies such as education on adequate nutritional intake during pregnancy, food fortification with iron and folic acid supplementations, malaria, and worm control have been put in place to contribute to the prevention of maternal anaemia (13).

The study on factors influencing anaemia in pregnancy in Ghana is important for several reasons. Firstly, anaemia is a major public health concern in Ghana, with high prevalence rates among pregnant women. According to the 2014 Ghana Demographic and Health Survey (GDHS), the prevalence of anaemia among pregnant women in Ghana was 63%, indicating a significant burden of the disease (14). Anaemia during pregnancy is also associated with adverse maternal and foetal outcomes, such as preterm delivery, low birth weight, and maternal mortality (15). Understanding the factors that contribute to anaemia during pregnancy in Ghana can inform the development of effective interventions to reduce the burden of the disease.

However, there is limited research on the factors influencing anaemia during pregnancy in Ghana. Previous studies have focused on individual risk factors, such as iron deficiency and dietary habits, but there is a need for a more comprehensive understanding of the social and environmental factors that contribute to anaemia during pregnancy. According to a study by Tawiah et al. (16), social determinants of health, such as educational level and occupation, are important predictors of anaemia during pregnancy in Ghana. Therefore, a study that examines a broad range of factors, including social and environmental determinants, can provide a more comprehensive understanding of the factors that contribute to anaemia during pregnancy in Ghana. Identifying the factors that contribute to anaemia during pregnancy in Ghana has practical implications for the development of effective interventions. According to the World Health Organization (2), interventions that address multiple factors, such as iron supplementation, nutrition education, and antenatal care, are more effective in reducing the burden of anaemia during pregnancy. This study investigated the factors influencing anaemia among pregnant women attending antenatal clinics (ANCs) in the Adaklu District.

## Methods

### Study setting

The research was carried out in five health centres in the Adaklu District (Waya, Ahunda, Nutifafa, Soda, and Helekpe).

### Study design

The study was a quantitative one in which the factors associated with anaemia among pregnant women were investigated. A prospective study design was used to conduct this research in the Adaklu District where baseline data were obtained from participants’ records and participants were followed up until their third trimester. All expectant women who voluntarily signed the consent forms and were willing to participate in the study had their first trimester data collected from their ANC health records. In order to acquire further level data and track the evolution of anaemia, participants in their first trimesters were followed up for both the second and third trimesters while those in their second trimester were followed up for their third trimester haemoglobin level data. This design was deemed appropriate since it is less susceptible to research biases than other observational studies. Prospective studies are more robust than retrospective studies in establishing the temporal relationship between exposure and outcome (17). This is because prospective studies collect data on exposure and outcome over time, thus reducing the potential for recall bias. In the context of anaemia among pregnant women, a prospective design is also appropriate because it allows for the collection of data on risk factors for anaemia during pregnancy, such as dietary habits and antenatal care attendance, which may change over time. A cohort design means that follow-up is done on a homogenous group of people at a point in time. This study took place from May 2019 to August 2020.

### Sampling procedure

The sampling procedure for the study was census. The researcher used voluntary participation to sample the participants. This technique is a quantitative method that attempts to collect information from all eligible participants in a defined population; hence, all pregnant women attending ANCs in the district were sampled for the study. Regarding the sample size, the study used census to collect data from all registered pregnant women who were attending the antenatal clinics at Adaklu health centres and were available during data collection. Pregnant women with bleeding disorders were excluded from the study. The total number of registrants in the Adaklu District over the period of 3 months was 169. Out of the 169 pregnant women, 19 were unwilling to participate. The reasons were unknown; hence, 150 pregnant women were sampled for the study.

### Data collection

Data were gathered using a questionnaire designed to elicit information from the participants. Data were collected on the prevalence and knowledge level of pregnant women about anaemia, perceived barriers to maintaining an appropriate haemoglobin level, health education given by health professionals to the pregnant women, and factors that determine anaemia among pregnant women. Secondary data on haemoglobin levels were obtained from participants’ folders.

### Ethics approval

Ethical clearance for the study was obtained from the University of Cape Coast Institutional Review Board (UCCIRB/CES/2019/14). Permission was obtained from the district health directorate, which granted access to the health centres for the data collection. Participants were assured of no risks in participating in the study, anonymity was ensured, and informed consent was also obtained from the participants before data collection.

### Data analysis

The data were coded and processed with SPSS version 21.0. Percentages and frequencies were used to analyse the data on prevalence, knowledge, and barriers to maintaining appropriate haemoglobin levels. To ascertain the factors that are associated with maternal anaemia during pregnancy in the Adaklu District, binary logistic regression was used to determine the factors that predicted the development of anaemia in the district (18). The dependent variable was anaemia status dichotomised into anaemic and non-anaemic. The independent variables were categorical in nature (age, parity, gravida, educational levels, marital status, religion, income level, and occupation).

## Results

This section highlights the number of pregnancies among the participants, their demographic characteristics, and their ANC attendance. It also presents the prevalence of anaemia among the participants in the first, second, and third trimesters of pregnancy. These results provide insight into the burden of anaemia among pregnant women in a rural setting of the Adaklu District and the need for interventions to address this issue.

Table 1 represents the socio-economic characteristics of 150 study participants. The mean age of the participants was 27.1 (SD = 3.6). This implies that in average, the participants were in their youthful age. Those who married comprised 76%, while 2.7% were divorced.

**Table 1:** Socio-economic and demographic profiles of the study participants.

Characteristics	*n* = 50	%
Marital status		
Married	114	76.0
Divorced	4	2.7
Single	32	21.3
Number of pregnancies		
2–3	147	98.0
4 or more	3	2.0
Number of children		
No child	37	24.7
1–3 children	77	51.3
4–6 children	36	24.0
Religion		
Christian	115	76.7
Islam	19	12.7
Traditional	16	10.6
Educational levels		
Non-formal	58	38.7
Primary	66	44.0
Vocational	8	5.3
Technical	11	7.3
Secondary	7	4.7
Income in Ghana cedi		
Less than 300	96	64.0
300–500	37	24.7
501–700	9	6.0
701–900	2	1.3
900 and above	6	4.0
Occupation		
Farming	80	53.3
Unemployed	17	11.3
Public servant	12	8.0
Trading	35	23.3
Student	6	4.0
Number of ANC visits		
2–3	85	56.6
4 or more	65	43.4

The median income was about 1,000.00 Ghana cedi monthly.

In terms of the participants’ pregnancy history, all women had previously given birth. In addition, the study revealed that most participants were married Christians who worked as farmers. In terms of ANC visits, 57% of the participants attended two to three times, while the remaining participants attended four or more times.

The study found that 75% (*n* = 113) of the participants were anaemic during the first trimester, 81% (*n* = 121) during the second trimester, and 79% (*n* = 118) during the third trimester. The overall prevalence of anaemia among pregnant women over the three trimesters was 78.3%. With the high prevalence of anaemia among the women, the study further explored maternal knowledge of anaemia. The results indicated that 92% (*n* = 138) of the pregnant women had excellent knowledge of anaemia, while only 8% (*n* = 12) had good knowledge.

The study further investigated barriers to maintaining appropriate haemoglobin levels during pregnancy in the district. Table 2 shows the perceived barriers to maintaining an appropriate haemoglobin level among pregnant women in the Adaklu District. Out of the 150 pregnant women in the district, 92% (*n* = 138) agreed that their partners supported them financially, while 8% (*n* = 12) disagreed with partner support (Table 3). Furthermore, 57.3% (*n* = 86) of the pregnant women agreed that in their community, pregnant women are not allowed to take snails, while 42.7% (*n* = 64) of the pregnant women disagreed. Also, 91.3% (*n* = 137) of the pregnant women agreed that the distance to the health facility was very far, while 8.7% (*n* = 13) of the pregnant women disagreed. The barriers to maintaining appropriate haemoglobin levels, as found in the study, were non-intake of eggs, snails, and antimalarial drugs during ANC visits. Secondly, distance from the healthcare facilities is another potential challenge for the pregnant women in maintaining haemoglobin levels.

**Table 2:** Perceived barriers in maintaining appropriate haemoglobin level among pregnant women.

Statements	Adaklu district			
	Agreed		Disagreed	
	*N*	%	*n*	%
Whether partner supports me financially	138	92.0	12	8.0
My partner allows me to go for antenatal visits	130	88.7	20	13.3
I am given iron pills for free at the clinic	137	91.3	13	9.7
I do not take folic acid regularly	13	9.7	137	91.3
In my community, pregnant women do not take eggs	48	32.0	102	68.0
In my community, pregnant women do not take snail	86	57.3	64	42.7
The attitude of the health workers is encouraging	115	77.0	35	23.0
I do not have a valid National Health Insurance card	22	14.7	128	85.3
I have taken deworming during pregnancy	115	77.0	35	23.0
I was given antimalarial drugs during ANC visits	102	68.0	48	32.0
My religion accepts blood transfusion	113	75.3	37	24.7
Antenatal visit is not beneficial to me	12	8.0	138	92.0
The distance to the health facility is very far	137	91.3	13	8.7

**Table 3:** Components of health education provided to pregnant women in the study area.

Parameters	Yes		No	
	*n*	%	*N*	%
Have you been given health talk on how to prevent malaria?	147	98.0	3	2.0
Have you been given health talk on cleanliness?	144	96.0	6	4.0
Have you been given health talk on the benefits of good nutrition?	143	95.3	7	4.7
Have you been give health talk on the benefits of regular medical check-ups?	143	95.3	7	4.7
Have you been given health talk on adequate rest?	139	92.7	11	7.3
Have you been given health talk on anaemia at ANC?	137	91.3	13	8.7
Have you been given health talk on birth preparedness?	132	88.0	18	12.0
Have you been given health talk on family planning?	127	84.7	23	15.3
Have you been given health talk on effects of alcohol use?	131	87.3	19	12.7
Have you been given health talk on how to prevent worm infestation?	134	89.3	16	10.7

The study further explored the components of health education given to pregnant women during ANC visits. Most of the pregnant women, 98% (*n* = 147), agreed that they received education on malaria when they attended an antenatal clinic in the district.

To find out factors associated with anaemia levels among pregnant women, a binary logistic regression was used to determine the factors that are associated with anaemia among pregnant women in the district. Tables 4–6 present results on the determinants of anaemia status during pregnancy within the first, second, and third trimester in the Adaklu District, respectively. Univariate and multivariate analyses were used to examine the determinants of anaemia status during pregnancy. The variables used in the model were the age of the respondents, marital status, number of pregnancies, number of children, religion, educational level, income, and occupation; hence, they were all included in the model. The discussion of the results was done based on each trimester. The results show that women with four or more pregnancies (OR = 0.77, 95% CI = 0.36–1.65) were more likely to have anaemia in the first trimester, compared to women who were pregnant for two to three times. Also, anaemia is more likely to affect pregnant women who have had one to three children (OR = 2.71, 95% CI = 0.90–8.19) and four to six children (OR = 0.50, 95% CI = 0.16–1.61) under the first trimester than those with no children.

**Table 4:** Determinants of anaemia status during pregnancy in the Adaklu district (trimester 1).

Characteristics	*n* (%) with outcome	Univariate OR (95% CI)	*P*-value	Multivariate OR (95% CI)	*P*-value
Age (years)					
<26	57 (38.0)		0.356		0.541
26–35	78 (52.0)	1.73 (0.34–8.75)		2.07 (0.40–10.70)	
≥36	15 (10.0)	1.18 (0.24–5.92)		2.08 (0.54–8.04)	
Marital status					
Married	114 (76.0)		0.562		0.849
Divorced	4 (2.7)	0.93 (0.41–2.13)		0.90 (0.29–2.74)	
Single	32 (21.5)	0.52 (0.07–4.24)		0.58 (0.06–5.63)	
Number of pregnancies					
2–3	147 (98)		0.502		0.429
4 or more	3 (2)	0.77 (0.36–1.65)		0.53 (0.17–1.63)	
Number of children					
No child	37 (24.7)		0.038		0.566
1–3 children	77 (51.3)	2.71 (0.90–8.19)		1.77 (0.37–8.50)	
4–6 children	36 (24.0)	0.50 (0.16–1.61)		2.24 (0.63–7.94)	
Religion					
Christian	115 (76.7)		0.550		0.186
Islam	19 (12.7)	1.56 (0.33–7.41)		2.89 (0.76–11.05)	
Traditional	16 (12.7)	1.31 (0.19–9.02)		2.28 (0.48–10.83)	
Educational levels					
Non-formal	58 (38.7)		0.807		0.008
Primary	66 (44.0)	0.46 (0.08–2.74)		2.09 (0.15–29.79)	
Vocational	8 (5.3)	0.61 (0.11–3.52)		0.76 (0.06–10.62)	
Technical	11 (7.3)	0.36 (0.03–5.11)		3.03 (0.16–56.36)	
Secondary	7 (4.7)	0.25 (0.02–3.47)		0.17 (0.01–3.00)	
Income (GHC)					
300–500	133 (88.7)		0.523		0.281
>500	17 (11.3)	1.65 (0.35–7.70)		0.47 (0.09–2.59)	
Occupation					
Farming	80 (53.3)		0.308		0.358
Unemployed	17 (11.3)	0.97 (0.11–9.00)		1.11 (0.15–8.31)	
Public servant	12 (8)	0.31 (0.02–5.96)		1.70 (0.20–14.54)	
Trading	35 (23.3)	1.00 (0.07–13.87)		0.18 (0.01–3.76)	
Student	6 (4)	1.73 (0.18–16.87)		0.61 (0.08–4.78)	

**Table 5:** Determinants of anaemia status during pregnancy in the Adaklu district (trimester 2).

Characteristic	*n* (%) with outcome	Univariate OR (95% CI)	*P*-value	Multivariate OR (95% CI)	*P*-value
Age (years)					
<26	57 (38.0)		0.068		0.252
26–35	78 (52.0)	2.26 (0.68–7.52)		2.82 (0.55–14.50)	
≥36	15 (10.0)	1.07 (0.35–3.30)		1.54 (0.41–5.81)	
Marital status					
Married	114 (76.0)		0.639		0.461
Divorced	4 (2.7)	0.78 (0.33–1.86)		1.61 (0.50–5.19)	
Single	32 (21.5)	0.39 (0.05–3.22)		0.48 (0.05–4.54)	
Number of pregnancies					
2–3	147 (98.0)		0.587		0.426
4 or more	3 (2.0)	0.81 (0.37–1.76)		0.42 (0.14–1.31)	
Number of children					
No child	37 (24.7)		0.282		0.195
1–3 children	77 (51.3)	1.76 (0.64–4.84)		2.79 (0.55–14.14)	
4–6 children	36 (24.0)	1.05 (0.46–2.39)		2.06 (0.58–7.26)	
Religion					
Christian	115 (76.7)		0.381		0.133
Islam	19 (12.7)	1.71 (0.59–4.94)		2.90 (0.75–11.15)	
Traditional	16 (12.7)	1.66 (0.42–6.72)		1.86 (0.39–8.78)	
Educational levels					
Non-formal	58 (38.7)		0.004		0.003
Primary	66 (44.0)	7.86 (1.37–45.06)		12.11 (0.77–190.63)	
Vocational	8 (5.3)	5.36 (0.96–29.91)		4.63 (0.32–68.06)	
Technical	11 (7.3)	4.17 (0.47–36.74)		4..77 (0.25–92.32)	
Secondary	7 (4.7)	2.08 (0.28–15.77)		1.69 (0.10–28.00)	
Income (GHC)					
300–500	133 (88.7)		0.065		0.982
>500	17 (11.3)	2.62 (0.94–7.27)		0.83 (0.18–3.80)	
Occupation					
Farming	80 (53.3)		0.572		0.828
Unemployed	17 (11.3)	1.04 (0.18–6.04)		0.56 (0.07–4.60)	
Public servant	12 (8.0)	3.75 (0.40–35.54)		1.97 (0.18–21.47)	
Trading	35 (23.3)	0.36 (0.04–2.77)		0.53 (0.03–11.31)	
Student	6 (4.0)	0.96 (0.15–6.01)		0.62 (0.07–5.37)	

**Table 6:** Determinants of anaemia status during pregnancy in the Adaklu district (trimester 3).

Characteristic	*n* (%) with outcome	Univariate OR (95% CI)	*P*-value	Multivariate OR (95% CI)	*P*-value
Age (years)					
<25	57 (38.0)		0.047		0.198
26–35	78 (52.0)	0.17 (0.04–0.67)		0.12 (0.02–0.71)	
≥36	15 (10.0)	0.17 (0.05–0.67)		0.11 (0.02–0.55)	
Marital status					
Married	114 (76.0)		0.284		0.523
Divorced	4 (2.7)	1.50 (0.67–3.35)		0.96 (0.33–2.77)	
Single	32 (21.5)	0.56 (0.05–5.97)		0.43 (0.04–5.19)	
Number of pregnancies					
2–3	147 (98.0)		0.760		0.410
4 or more	3 (2.0)	0.88 (0.40–1.97)		2.16 (0.74–6.27)	
Number of children					
No child	37 (24.7)		0.049		0.104
1–3 children	77 (51.3)	0.38 (0.15–1.99)		0.31 (0.07–1.38)	
4–6 children	36 (24.0)	0.83 (0.38–1.83)		0.82 (0.25–2.68)	
Religion					
Christian	115 (76.7)		0.426		0.353
Islam	19 (12.7)	0.92 (0.32–2.65)		0.98 (0.27–3.55)	
Traditional	16 (12.7)	2.20 (0.57–8.57)		2.73 (0.59–12.76)	
Educational levels					
Non-formal	58 (38.7)		0.894		0.001
Primary	66 (44.0)	1.16 (0.24–5.66)		0.12 (0.01–2.67)	
Vocational	8 (5.3)	0.98 (0.20–4.74)		0.15 (0.01–3.45)	
Technical	11 (7.3)	1.33 (0.17–10.25)		0.22 (0.01–5.53)	
Secondary	7 (4.7)	1.1 (0.16–7.51)		0.24 (0.01–6.28)	
Income (GHC)					
300–500	133 (88.7)		0.759		0.908
>500	17 (11.3)	1.17 (0.42–3.27)		2.54 (0.47–13.51)	
Occupation					
Farming	80 (53.3)		0.193		0.724
Unemployed	17 (11.3)	2.00 (0.35–11.54)		1.40 (0.19–10.47)	
Public servant	12 (8.0)	1.40 (0.20–9.87)		1.34 (0.16–10.11)	
Trading	35 (23.3)	1.00 (0.13–7.10)		0.28 (0.01–8.31)	
Student	6 (4)	1.33 (0.24–8.29)		0.79 (0.10–6.27)	

In the first trimester, under the multivariate outcome, the level of education among pregnant women was a predictor of anaemia. The level of education identified by the pregnant women were primary (OR = 2.09, 95% CI = 0.15–29.79), vocational (OR = 0.76, 95% CI = 0.06–10.62), technical (OR = 3.03, 95% CI = 0.16–56.36), and secondary education (OR = 0.17, 95% CI = 0.01–3.00). Pregnant women who had vocational and secondary education are more likely to experience anaemia than those with tertiary education. Again, pregnant women without formal education were also discovered to experienced anaemia.

Table 5 shows that educational level is associated with anaemia in the second trimester both in univariate and multivariate analyses. The results indicate that higher educational level decreases the odds of anaemia in the population such that participants with primary education (OR = 7.86, 95% CI = 1.37–45.06), vocational (OR = 5.36, 95% CI = 0.96–29.91), technical (OR = 4.17, 95% CI = 0.47–36.74), and secondary education (OR = 2.08, 95% CI = 0.28–15.77) were at a higher risk of anaemia in the study area than pregnant women with tertiary education. Similarly, in the multivariate analysis, higher educational level decreases the odds of anaemia in the population such that participants with primary education (OR = 12.11, 95% CI = 0.77–190.63), vocational (OR = 4.63, 95% CI = 0.32–68.06), technical (OR = 4.77, 95% CI = 0.25–92.32), and secondary education (OR = 1.69, 95% CI = 0.10–28.00).

Also, in the univariate analysis, it shows that the chances of anaemia in the second trimester decreases with age such that women aged 26–35 years (OR = 2.26, 95% CI = 0.68–7.52) and ≥36 years (OR = 1.07, 95% CI = 0.35–3.30) were less likely to have anaemia than younger respondents. In addition, women with higher incomes than >500 GHC (OR = 2.62, 95% CI = 0.94–7.27) were less likely to have anaemia as their odd ratio increases.

Results on the third trimester of pregnancy revealed that pregnant women between the ages of 26 and 35 (OR = 0.17, 95% CI = 0.04–0.67) and those over 36 (OR = 0.17, 95% CI = 0.05–0.67) were more likely to develop anaemia in the third trimesters than those below the ages of 26 years. Also, the number of children was found to be significant as women with one to three children (OR = 0.38, 95% CI = 0.15–1.99) and four to six children (OR = 0.83, 95% CI = 0.38–1.83) were more likely to have anaemia than women without any pregnancy.

Finally, in the third trimester, under the multivariate outcome, the level of education among pregnant women significantly is associated with anaemia. The level of education identified by the pregnant women were primary (OR = 0.12, 95% CI = 0.01–2.67), vocational (OR = 0.15, 95% CI = 0.01–3.45), technical (OR = 0.22, 95% CI = 0.01–5.53) ,and secondary education (OR = 0.25, 95% CI = 0.01–6.28). The results from the levels of education revealed that pregnant women who had primary, vocational, technical, and secondary education are more likely to experience anaemia than those with tertiary education.

## Discussion

The overall prevalence of maternal anaemia in the Adaklu District was 78%. The findings revealed that anaemia occurred in all three trimesters of pregnancy, with the highest occurrence in the second trimester. The findings on the prevalence of anaemia among pregnant women indicated that there is a high prevalence of anaemia in pregnancy in the Adaklu District. This high prevalence of anaemia means there is a possibility of an increase in the risk of premature birth, low birth weight babies, and postpartum depression among these women. The plausible reasons for this finding could be irregular intake or non-intake of iron supplements with fruits rich in vitamin C (orange, guava, and pineapple) among the pregnant women. The prevalence of 78% of anaemia suggests that a large proportion of pregnant women in the district are at risk of experiencing adverse maternal and foetal outcomes, such as preterm delivery, low birth weight, and maternal mortality. This high prevalence of maternal anaemia in the Adaklu District is concomitant with the findings of the Ghana Demographic Health Survey (19), which revealed that out of the 25 districts in the Volta Region, the Adaklu District had the highest percentage of 72% in 2016, while the Nkwanta North District was second in the same year with a percentage of 68.8%. Several studies have reported a high prevalence of anaemia among pregnant women in Ghana. For example, a study by Asare et al. (20) found that 73.5% of pregnant women in the Ashanti region of Ghana were anaemic. Another study by Boadu et al. (21) found a prevalence of 63.9% among pregnant women attending antenatal clinics in the Upper West region of Ghana. The high prevalence of anaemia among pregnant women in Ghana is attributable to several factors. One of the main factors is iron deficiency, which is the leading cause of anaemia in pregnancy. Studies have shown that inadequate dietary iron intake is common among pregnant women in Ghana, particularly in rural areas where poverty is high (22, 23). In addition, infections such as malaria and hookworm infestation can contribute to anaemia in pregnancy (2, 22). Other factors contributing to the high prevalence of anaemia in pregnancy in Ghana include poor antenatal care attendance, a lack of knowledge about the importance of nutrition in pregnancy, and limited access to iron supplements and other interventions (20, 21, 23). This indicates that the anaemia in pregnancy situations has not changed in the district despite several health education sessions at ANCs. There is a need for immediate interventions to curb the problem and reduce its consequences for both the mother and the child.

The findings also revealed that pregnant women in the Adaklu District have a high level of knowledge about anaemia. The high knowledge levels on the nature of anaemia, its signs and symptoms, and prevention measures mean the pregnant women understood the health education they received at the ANC. In general, the increased level of knowledge on anaemia should have led to a reduction in the prevalence of the disease in the district. Unfortunately, the knowledge did not commensurate with the prevalence of anaemia in the district. This could be because knowledge may not always be translated into action, or some pregnant women may not apply their knowledge due to cultural barriers. Cultural norms may be responsible for the high prevalence of anaemia, as pregnant women are prohibited from consuming snails, which are rich in protein and could boost haemoglobin levels. The findings on the knowledge level of pregnant women on anaemia in the Adaklu District support the findings of Yadav et al. (24) that knowledge did not contribute to the prevalence level.

The findings on perceived barriers to maintaining an appropriate haemoglobin level among pregnant women in the Adaklu District revealed that some pregnant women did not take eggs or snails due to cultural beliefs. Cultural beliefs and practises that limit women's access to nutritious foods and discourage them from seeking medical care during pregnancy can contribute to the high prevalence of anaemia among pregnant women in Ghana (22, 23). Another potential barrier is the long distance to the health facility. The implications of the finding on perceived barriers to maintaining an appropriate haemoglobin level among pregnant women in the Adaklu Districts suggest that socio-cultural beliefs increase the possibility of the pregnant women developing anaemia. In relation to the pregnant women who took dewormers during pregnancy, the outcome is in line with the views of Jufar and Zewde (25) that worm infection is the result of women's craving for soil during pregnancy and may contribute to anaemia. An antenatal visit is beneficial to pregnant women. The long distance to the health facility is a potential challenge. This means that ANC services should be extended to communities in order to reduce the maternal burden of long travels for healthcare. This supports the views of Nisar et al. (26), who endorsed the need to formulate and implement policies like community awareness campaigns to encourage the availability of healthcare professionals and encourage pregnant women to access antenatal services in their communities. In addition, it was identified that most of the pregnant women were financially supported by their partners. Again, their partners allowed them to go for antenatal visits.

Furthermore, the findings revealed that health education is received well by the participants at the ANC. The high endorsement of health talk received at ANCs on the benefits of good nutrition and regular medical check-ups is in support of the earlier observation by Adam (27) in the Central Region, where he found the knowledge level of pregnant women on good nutrition to be about 90%. The consistency of the finding with that of Adam (27) could be that ANC visits are not only for medication but also serve as a contact point for health promoters to interact with pregnant women. Mulepati and Chaudhary (28), Amoakoh-Coleman et al. (29), and the WHO (30) explained the effectiveness of ANC visits by stating that the visits help recognise and distinguish between pregnant women who need special care and those who need normal care. Dhange et al. (31) underscored the reasons for ANC visits for pregnant women even if they have no complications by stating that the principles of ANC are to give health education, conduct screenings, treat minor ailments, and make referrals. That is, ANC visits are at the heart of health education, and pregnant women can best be educated if they are present at the health facilities. In essence, it is during the health talks on ANC that the health promoters can help the pregnant women understand the need to be regulars at the health facility even if they feel they are not ill.

Regarding factors associated with anaemia levels among pregnant women, educational level, number of pregnancies, number of children, and maternal age were significant determinants of anaemia status in the Adaklu District. This implies that healthcare providers should continuously educate pregnant women. The results of several studies have shown that educational level is a significant factor in the prevalence of anaemia in Ghana. For example, a study by Abizari et al. (32) found that the prevalence of anaemia was higher among school children with lower educational levels. The authors suggest that this may be due to poor dietary habits and inadequate knowledge of good nutrition among children. Similarly, a study by Mensah et al. (33) found that women with lower educational levels had a higher prevalence of anaemia compared to those with higher educational levels. The authors suggest that this may be due to a lack of knowledge about the importance of good nutrition and the need for iron-rich foods. Likewise, Ługowska and Kolanowski (34) pointed out that education is essential for spreading awareness about healthy eating, especially during pregnancy. However, Novivanti et al. (35) revealed that less-educated pregnant women have a lower probability of being anaemic than highly educated ones.

The findings indicated that women who had four or more pregnancies were more likely to develop anaemia in the first trimester. Women who are pregnant already feel more tired than usual because of hormonal changes and a higher demand for nutrients during pregnancy. On the other hand, pregnant women who have given birth to four to six children have a high probability of developing anaemia. Pregnancy imposes an additional burden on the body, particularly for mothers who have already had several children. By lowering the blood's ability to deliver oxygen, anaemia worsens maternal susceptibility. This may make it harder for mothers to handle the physical and emotional strains of carrying another child since it may increase the physical demands of pregnancy. Again, the risk of problems during pregnancy grows for both the expectant mother and the growing foetus. Due to the cumulative demands of prior pregnancies, the bodies of women who have had many pregnancies may already be lacking in vital nutrients like proteins and minerals like iron. These difficulties can lead to maternal and child health problems such as preterm birth, low birth weight, postpartum haemorrhage, and maternal morbidity. The findings of the study are in agreement with Ramulondi et al. (36), who asserted that women who have had several pregnancies may experience postpartum recovery issues due to anaemia. Again, the number of children given birth to by pregnant women had a significant influence on anaemia. Anaemia is more likely to affect pregnant women who have had four to six children under the first trimester. This implies that these women are going through the physical and physiological changes related to pregnancy. Similarly, Stevens et al. (6) reported that anaemia lowers the blood's ability to carry oxygen, which can cause weakness and fatigue during the first trimester of pregnancy. The findings during the third trimester of pregnancy revealed that pregnant women between the ages of 26 and 35 and those over 36 are more likely to develop anaemia in the third trimester. This implies that pregnant women with an increase in age are more likely to develop anaemia. Women's natural iron reserves naturally decline with age. One of the factors contributing to anaemia during pregnancy is iron deficiency. Di Renzo et al. (37) stated that pregnancy-related anaemia is more likely to occur if the body's iron stores are already low before the pregnancy. Again, chronic diseases such as kidney disease, autoimmune diseases, and gastrointestinal issues are more common in older people. Some of these diseases increase the risk of anaemia during pregnancy by interfering with iron intake, utilisation, or red blood cell formation.

Overall, the educational levels of the pregnant women were significant determinants of anaemia in all three trimesters. The findings showed that anaemia is more common in pregnant women with less education in the second trimester. For the foetus's growth and development as well as their health, pregnant women need higher levels of nutrients such as iron and folate. Women may fail to get enough of these vital nutrients if they are not well informed on the importance of a nutritious diet during pregnancy, which increases their chance of developing anaemia. Again, cultural influences can affect prenatal dietary practises and attitudes. In some societies, dietary limitations or customs during pregnancy may lead to insufficient nutrient intake. For instance, cultural attitudes could encourage dietary restrictions that restrict the diversity of nutrients available to pregnant women or prohibit the use of foods high in iron. Without education on the value of healthy nutrition during pregnancy, women may follow cultural customs that raise their risk of anaemia. Therefore, efforts to address the problem of maternal anaemia should focus on health education and promoting healthy lifestyles among individuals with lower educational levels. These findings also underscore the need for targeted interventions to address factors and reduce the burden of anaemia in the country.

## Conclusions

Anaemia is a significant health problem among pregnant women in Ghana. The high prevalence of anaemia is largely attributed to factors such as poor nutrition, parasitic infections, and inadequate antenatal care. The consequences of anaemia during pregnancy are far-reaching and can have adverse effects on both the mother and child. To reduce the burden of anaemia among pregnant women in Ghana, there is a need for a multi-faceted approach that addresses the underlying factors contributing to anaemia in pregnancy in terms of diets, ANC attendance, and socio-cultural beliefs. Iron supplementation, dietary interventions, and deworming are effective interventions to reduce the prevalence of anaemia. However, these interventions need to be combined with efforts to improve antenatal care attendance and health education to promote good nutrition during pregnancy. It is also essential to address the broader social determinants of health, such as poverty and gender inequality, which affect women's access to resources and contribute to poor nutrition. To improve the health outcomes of pregnant women and their babies, there is a need for concerted efforts by the government, healthcare providers, and the community at large. The government can prioritise the provision of accessible antenatal care services and support programmes aimed at reducing poverty and improving the economic status of women. Healthcare providers can also promote health education and counselling on nutrition during pregnancy, in addition to iron supplementation and deworming interventions. Overall, reducing the burden of anaemia among pregnant women in Ghana requires a comprehensive approach that addresses the underlying factors contributing to anaemia in pregnancy. It is essential to continue research on effective interventions and to work collaboratively to improve the health outcomes of pregnant women in Ghana.

### Strengths of the study

The major strength of this study was that it identified risk factors that contribute to anaemia among pregnant women attending antenatal clinics at health centres in a rural setting in Ghana's Adaklu District. The validity and reliability of the research instrument were ensured by pregnant women who visited the antenatal clinics for the first time during the study. The instrument's reliability was assessed using the Kuder–Richardson Formula (KR21), and after adding four extra items to improve the reliability, the KR21 value was found to be 0.815, indicating a very good level of reliability.

### Limitations of the study

The study focused on pregnant women who attended ANCs in the Adaklu District. The findings of the study could not be generalised because pregnant women who did not attend ANCs were not part of the study. Secondly, the researchers did not check the haemoglobin levels themselves, so they solely relied on the haemoglobin levels recorded on the pregnant women's antenatal booklets. In spite of its limitations, the study provided vital information on the factors influencing maternal anaemia in the Adaklu District.

## Data Availability

The raw data supporting the conclusions of this article will be made available by the authors, without undue reservation.
